# A Brief Review of Non-invasive Monitoring of Respiratory Condition for Extubated Patients with or at Risk for Obstructive Sleep Apnea after Surgery

**DOI:** 10.3389/fmed.2017.00026

**Published:** 2017-03-08

**Authors:** Xuezheng Zhang, Mahmoud Attia Mohamed Kassem, Ying Zhou, Muhammad Shabsigh, Quanguang Wang, Xuzhong Xu

**Affiliations:** ^1^Anesthesiology Department, The First Affiliated Hospital of Wenzhou Medical University, Wenzhou, China; ^2^Anesthesiology Department, Wexner Medical Center of Ohio State University, Columbus, OH, USA; ^3^Pulmonary Medicine, The First Affiliated Hospital of Wenzhou Medical University, Wenzhou, China

**Keywords:** obstructive sleep apnea, surgery, monitoring, oximetry, capnography, photoplethysmography, respiratory volume monitor

## Abstract

Obstructive sleep apnea (OSA) is one of the important risk factors contributing to postoperative airway complications. OSA alters the respiratory physiology and increases the sensitivity of muscle tone of the upper airway after surgery to residual anesthetic medication. In addition, the prevalence of OSA was reported to be much higher among surgical patients than the general population. Therefore, appropriate monitoring to detect early respiratory impairment in postoperative extubated patients with possible OSA is challenging. Based on the comprehensive clinical observation, several equipment have been used for monitoring the respiratory conditions of OSA patients after surgery, including the continuous pulse oximetry, capnography, photoplethysmography (PPG), and respiratory volume monitor (RVM). To date, there has been no consensus on the most suitable device as a recommended standard of care. In this review, we describe the advantages and disadvantages of some possible monitoring strategies under certain clinical conditions. According to the literature, the continuous pulse oximetry, with its high sensitivity, is still the most widely used device. It is also cost-effective and convenient to use but has low specificity and does not reflect ventilation. Capnography is the most widely used device for detection of hypoventilation, but it may not provide reliable data for extubated patients. Even normal capnography cannot exclude the existence of hypoxia. PPG shows the state of both ventilation and oxygenation, but its sensitivity needs further improvement. RVM provides real-time detection of hypoventilation, quantitative precise demonstration of respiratory rate, tidal volume, and MV for extubated patients, but no reflection of oxygenation. Altogether, the sole use of any of these devices is not ideal for monitoring of extubated patients with or at risk for OSA after surgery. However, we expect that the combined use of continuous pulse oximetry and RVM may be promising for these patients due to their complementary function, which need further study.

## Introduction

Complications of the airway are major problems for patients in the early postoperative period (1). One of the independent risk factors associated with upper airway impairment including hypoventilation, high airway resistance, abnormal rhythm of breathing, and resulting hypoxia is obstructive sleep apnea (OSA). OSA strongly correlates with the increasing rates of obesity and is a frequent comorbidity affecting 2–26% of the general population (2–5). As for the surgical patients, the prevalence of OSA was reported to be even higher (24-41%) than general population (6). Furthermore, in the review by Zaremba, it was estimated that over 75% of the subpopulation of morbidly obese surgical patients may be affected by OSA (7).

Although OSA patients have the propensity of hypoventilation and hypoxia after surgery, the majority of OSA cases remain undiagnosed prior to surgery (8, 9). For this reason, several screening tools, such as the STOP-Bang, ASA checklist, sleep apnea clinical score, and Berlin questionnaire, were developed to assess the possible existence of an undiagnosed OSA and to provide risk stratification of OSA in surgical patients (10–12). A recent study showed that high-risk OSA patients had nearly fourfold increase in developing airway events in postanesthesia care unit (PACU) compared with low-risk OSA patients (13).

The partial or complete obstruction site in the upper airway of OSA patients may present at different levels, including the palatal region, tonsils, tongue base, epiglottis, hypopharynx, and lateral pharyngeal walls during sleep (14). Known or suspected sleep apnea, in the preoperative phase, may increase the risk of postoperative complications. Accordingly it increases the unplanned need for intensive care intervention, worsens surgical outcomes, and prolongs the length of hospital stay due to the high sensitivity of OSA patients to the residual effects of sedatives, neuromuscular agents, analgesics, and anesthetics on muscle tone of the upper airway (15–18). In addition, increased cardiac events, cerebrovascular impairment, and unexpected postoperative mortality were also documented (19–25). Therefore, early detection of respiratory impairment plays an important role in reducing mortality in non-intubated postoperative patients with possible OSA.

In the study by Chung et al., 120 patients without OSA were tested by a portable device, preoperatively. However, on postoperative night 1 and 3, they found out that 31 of those patients had apnea hypopnea index (AHI) >15, which defines the cutoff level of moderate OSA (26). That was implicated by the fact that surgery may have an impact on the incidence of OSA by altering sleep architecture, which possibly contributes to a greater degree of postoperative risk in OSA patients (10). The postsurgical period is characterized by disturbances in rapid eye movement phase of sleep, during which there are more occurrences of apneic episodes and desaturations (27). Further, postoperative analgesia with opioids contributes to pharyngeal muscle relaxation and attenuates the normal reaction to hypoxia and hypercapnia, thus aggravating respiratory status (28). Therefore, adequate monitoring of early respiratory impairment in OSA surgical patients is challenging.

Current clinically available monitoring devices for respiration in suspected or confirmed OSA patients include continuous pulse oximetry, capnography monitor, respiratory volume monitor (RVM), and photoplethysmography (PPG) in addition to clinical observation (Table 1). Clearly, effective management involving appropriate monitoring is pivotal to minimize postoperative risk for surgical patients with OSA. Until now, no consensus was reached for postoperative OSA monitoring in surgical patients when the trachea has been extubated and is no longer supported by a ventilator. This review briefly illustrates some possible monitoring strategies that we may use in clinical settings for extubated OSA patients after surgery under general anesthesia.

**Table 1:** **Features of different equipment**.

Equipment	Advantages	Disadvantages
Oximetry (Spacelab1600, USA)	Convenient, cost-effective, high sensitivity (95.6%) for desaturation	Low specificity (80.6%), lagging indication of respiratory abnormality, signal error; measures oxygenation, not ventilation; false alarm reduced signal quality in patients with poor peripheral perfusion

Capnography (Medtronic, USA)	Can be used in collaboration with SpO_2_ to reflect oxygenation and ventilation. High sensitivity (98%) and specificity (98%) for trachea intubated patients	EtCO_2_ can be influenced by hemodynamic disturbance, complexity in interpreting CO_2_ waveforms; maybe unable to detect hypoxemia; no report of sensitivity and specificity for extubated patients

PPG (Nonin Medical, USA)	Shows the condition of both ventilation and oxygenation with specificity of 91%	Low sensitivity (75%), multiple reasons such as motion, vasoconstrictor use, and heart rate change contribute to artifact of PPG

RVM (ExSpieon, USA)	Real-time detection of hypoventilation, quantitative precise demonstration of RR, TV, and MV for extubated patients, with sensitivity of 93%	May not work normally when the patients do not take supine position; with specificity of 86%

*SpO_2_, saturation of pulse oxygenation; EtCO_2_, end-tidal carbon dioxide; PPG, photoplethysmography; RVM, respiratory volume monitor; RR, respiratory rate; TV, tidal volume; MV, minute volume*.

## Continuous Pulse Oximetry

Continuous pulse oximetry is a routine method of oxygenation monitoring that measures the percentage of the oxygenated hemoglobin. By detecting hypoxemic episode, it is the primary objective indicator of respiratory compromise in clinical use. The use of pulse oximetry is recommended as a strategy to reduce complications of patients with OSA, such as short period hypoxemia, ICU transfer and death (29). Hypoxia detection by oximetry indicates the application of nasal CPAP or NIPPV (30). On the other hand, pulse oximetry is late to indicate respiratory depression, and as a result, its utility in evaluating the postoperative respiratory condition is limited. This inaccuracy of oximetry increases with increasing incidence of desaturation (31, 32). Meanwhile, Pedersen’s Cochrane review found no evidence of perioperative outcome improvement with routine use of pulse oximetry (33).

Pulse oximetry cannot detect reduced respiratory rate (RR), apnea, or exhaled carbon dioxide levels, which indicate ventilation impairment before dropping of SpO_2_ is displayed. Moreover, the supplemental oxygen may sometimes mask the existing respiratory depression and results in urgent events (34). Meanwhile, audible false alarms from signal error frequently occur during SpO_2_ monitoring and may misguide treatment or cause care providers to neglect identifying the real urgent circumstances that progress into crisis.

A previous study used SpO_2_ variability as a screening tool for OSA diagnosis and found that it has high sensitivity of 98% but only 46% specificity (35). The high sensitivity of SpO_2_ is beneficial to detect hypoxic event, but the low specificity probably reflects the inability to detect possible hypoventilation events when SpO_2_ is above 90%. Chung et al. recently demonstrated a 75% specificity of the oxygen desaturation index (hourly average number of desaturation episodes) to detect moderate and severe sleep-disordered breathing, giving another explanation why apnea or hypopnea may occur with normal SpO_2_ readings (36).

## Capnography/EtCO_2_ Measurement

Capnography detects the end-tidal carbon dioxide (EtCO_2_), which can provide insight into metabolic, circulatory, and respiratory activities. For extubated postanesthesia patients, the measurement of EtCO_2_ is done by placing a nasal cannula to obtain and analyze exhaled gas samples. EtCO_2_ may detect apneic episodes and respiratory depression indicated by reduced rate of respiration per minute. It is deemed superior to oximetry for earlier detection of an obstructed airway, opiate-induced apnea, or other airway problems in high-risk patients in the general care nursing unit.

According to Qadeer et al., capnography improves patient safety by reducing the frequency of hypoxia, severe hypoxemia, and apnea (37). OSA patients at risk for respiratory complications may benefit from EtCO_2_ monitoring, especially in the early postoperative period (38). However, extubated patients after surgery, especially those with OSA, are mouth breathers with high nasal airway resistance. Thus, obtaining accurate EtCO_2_ measurements *via* nasal cannula may be difficult. The difference between EtCO_2_ and PaCO_2_ will increase with fresh oxygen flow, a change more obvious in OSA patients (39, 40). For this reason, Kasuya et al. alternatively used mainstream and a nasal cannula that included an oral guide to reduce the inconsistency between EtCO_2_ and PaCO_2_, and it performed better than sidestream capnography for patients of OSA (41).

To date, there is no report that the RR reflected by capnography is incorrect. But it still has the problem that in many circumstances (41, 42), it may be easily dislodged by the patients. Otherwise, EtCO_2_ in intubated patients is technically reliable. Therefore, capnography must always be interpreted comprehensively together with other physiological parameters and clinical evaluation. Partial obstruction of the airway and mixing of exhaled air with ambient air may also display a normal EtCO_2_ value, whereas PaCO_2_ detectable by blood gas analysis is high.

## PPG-Derived Signal for RR

Photoplethysmography signal is composed of a respiratory modulation and variations associated with changing tissue blood volume of different origins. Analysis of the PPG waveform offers an alternative tool of non-invasive RR monitoring. The respiratory component signal is extracted from PPG. Based on the respiratory-induced intensity variation component, the RR and sleep apnea can be detected when intrathoracic pressure disappears (43).

By using the PPG signal principle, Addison et al. invented an algorithm designed to meet clinical needs by facilitating RR monitoring through a single probe. The probe provided continuous simultaneous monitoring of SpO_2_, heart rate, and RR for patients general care floor, including seven OSA patients (44). They found out that PPG-derived RR is comparable to the rate derived from EtCO_2_ waveform as reference. PPG-derived RR is more stable and less affected by motion-induced artifact and patient talk (43). In comparison to SpO_2_, PPG seems to be a better monitoring tool by using the combination of pulse oximetry with RR in a single sensor, thus obtaining earlier detection of respiratory compromise.

However, artifacts of PPG may still be present due to motion, vasomotor drugs and fluid administration, deep gasp, and heart rate changes (22). In a pilot study using neural network signal analysis, airway obstruction was identified by PPG-derived signals (Nonin Medical, USA) in a sample of extubated surgical patients prior to PACU arrival (75% sensitivity and 91% specificity) (45). When AHI > 68%, Romem et al. found that PPG demonstrated 70% sensitivity and 91% specificity compared to PSG (46). This low sensitivity is the main reason behind the limited application of PPG in clinical practice, since high sensitivity monitors are desired in the PACU to detect any possible adverse events. An 8% maximal error at detecting breaths in adult volunteers is another reason for the limited clinical application of PPG (47). With the improvement in neural network technology at combining all the contributing factors, the aforementioned sensitivity and specificity can be increased, and PPG may become a standard in future real-time monitoring.

Photoplethysmography-based respiratory monitoring shows the status of both ventilation and oxygenation, and further development is required to enable reliable assessment. At present, there are several studies about PPG for the evaluation of sleep apnea (48–50). However, there are limited reports of PPG as the special monitoring tool for postoperative OSA patients, although RR is regarded as a clinically important parameter for monitoring the respiratory condition (45, 51).

## RVM for Minute Volume

Minute volume (MV) and tidal volume (TV) data are displayed on standard ventilators when patients are intubated but are not available after extubation. A novel, Food and Drug Administration-approved and non-invasive RVM (ExSpieon, USA) is thoracic impedance-based respiratory monitor. It has recently become available to obtain objective numerical and curve expression through non-invasive measurement of MV, TV, and RR and to display continuous real-time respiratory curves in non-intubated patients. The MV is derived from changes in impedance associated with airflow in the lungs. These quantitative respiratory parameters, *via* a standardized set of thoracic electrode pads based on bioimpedance, can detect apneic and hypopneic episodes. Consequently, they can directly reflect respiratory compromise before hypercarbia and hypoxemia occur (52). Furthermore, it has a higher sensitivity than EtCO_2_ for monitoring of sedated patients in PACU (53). Earlier intervention can be initiated when low MV is identified compared to the capabilities of capnography (54). Physiologically, EtCO_2_ can reflect the abnormal ventilation condition of the patient but only after the occurrence of hypoventilation and hypercapnia. On the other hand, RVM can simultaneously detect and identify the duration of apneic and hypopneic episodes and the condition of recovery breaths, which are closely associated with the adequacy of ventilation. It has also the potential to markedly increase safety for patients in the postoperative period by more appropriate pain management, better decision-making regarding the time of PACU discharge, and decreasing respiratory compromise (55).

An over 24-h study by Voscopoulos et al. showed that RVM has similar accuracy and high correlation with spirometry in measuring MV, TV, and RR, based on comparison between RVM and ventilator measurements in both intubated and non-intubated patients (56, 57). It is also noteworthy that the patients’ average RR difference between the RVM measurements and the manual counting of individual breaths, by the technician, during the 24-ho study was 0.0 breaths/min, demonstrating that the RVM is extremely accurate in measuring RR (56). Furthermore, RVM can detect complete airway obstruction even when chest movement is present (58).

The RVM was not only able to measure the length and pattern of apneic episodes but also quantify the increase in the number and duration of such episodes as a response to drug administration (52, 58). In PACU, the quantitative measurements of MV from RVM can assist in decision-making to improve safety and optimize medication administration and care in patients with or at risk for OSA after extubation (54). Practically, the disadvantage of RVM is that it does not function normally when the patients are not placed in a supine position after surgery.

## Clinical Observations

In any patient, regardless of the existence of OSA, routine assessment for changes in ventilation adequacy, by direct clinical observation, seems to be subjective. Although continuous data are unavailable, this clinical comprehensive evaluation remains the obligation of care toward patients. A false reading, which may happen with each of the aforementioned equipment, is another explanation for demanding clinical judgment. Signs of respiratory distress, the ability to breathe deeply, upper airway muscle tone weakness, airway secretion, and, in more urgent circumstance, airway bleeding still depend on close observation by clinical staff (59). Further, none of the aforementioned types of monitoring methods remains well attached when the patient makes a movement or assumes an abnormal position. In a study involving a group of bariatric surgery patients, 30 of whom had severe OSA and were admitted in ICU for a 24-h postoperative observation, no relation between a high AHI and desaturations was found. Those findings could be explained by having one responsible nursing individual who may awaken the patients before the desaturation becomes severe (60).

## Conclusion

Management of patients with OSA after surgery is a major concern for perioperative care. It was shown that OSA is largely underestimated in the general population and often undiagnosed in surgical patients, resulting in higher postoperative morbidity. Early clinical recognition of hypoventilation can definitely help in designing a safer care plan.

Obstructive sleep apnea is characterized by apnea and its consequent respiratory impairment. Among the devices we mentioned, continuous pulse oximetry and capnography are valuable tools for detecting the consequences of apnea. PPG shows the condition of both ventilation and oxygenation and accurate detection of RR, but the sensitivity of PPG needs to be further improved. RVM may provide early detection of apneic episodes. Obviously, clinical monitoring is not suitable because it does not convey continuous data. We expect that the combined use of continuous pulse oximetry and RVM may be promising to provide potential safety for these patients, due to their complementary characteristics that need further investigation.

## Author Contributions

XZ, MK, YZ, MS, QW, and XX conducted a review of the literature. XZ, YZ, QW, and XX prepared the body of the manuscript.

## Conflict of Interest Statement

The authors declare that the review was conducted in the absence of any commercial or financial relationships that could be construed as a potential conflict of interest.

## References

[1] Fouladpour N Jesudoss R Bolden N Shaman Z Auckley D. Perioperative complications in obstructive sleep apnea patients undergoing surgery: a review of the legal literature. Anesth Analg (2016) 122(1):145–51. 10.1213/ANE.0000000000000841 26111263

[2] Memtsoudis S Liu SS Ma Y Chiu YL Walz JM Gaber-Baylis LK Perioperative pulmonary outcomes in patients with sleep apnea after noncardiac surgery. Anesth Analg (2011) 112(1):113–21. 10.1213/ANE.0b013e3182009abf 21081775

[3] Azagra-Calero E Espinar-Escalona E Barrera-Mora JM Llamas-Carreras JM Solano-Reina E. Obstructive sleep apnea syndrome (OSAS). Review of the literature. Med Oral Patol Oral Cir Bucal (2012) 17(6):e925–9. 10.4317/medoral.17706 22549673 PMC3505711

[4] Corso RM Gregoretti C Obstructive sleep apnea and anaesthesia: perioperative issues. Shortness Breath (2013) 2(2):72–9. 10.11138/sob/2013.2.2.072

[5] Chung F Memtsoudis SG Ramachandran SK Nagappa M Opperer M Cozowicz C Society of anesthesia and sleep medicine guidelines on preoperative screening and assessment of adult patients with obstructive sleep apnea. Anesth Analg (2016) 123(2):452–73. 10.1213/ANE.0000000000001416 27442772 PMC4956681

[6] Adesanya AO Lee W Greilich NB Joshi GP. Perioperative management of obstructive sleep apnea. Chest (2010) 138(6):1489–98. 10.1378/chest.10-1108 21138886

[7] Zaremba S Mojica JE Eikermann M. Perioperative sleep apnea: a real problem or did we invent a new disease? F1000Res (2016) 5. 10.12688/f1000research.7218.1 27006758 PMC4797892

[8] Auckley D Bolden N. Preoperative screening and perioperative care of the patient with sleep-disordered breathing. Curr Opin Pulm Med (2012) 18(6):588–95. 10.1097/MCP.0b013e3283589e6e 22990655

[9] Young T Evans L Finn L Palta M. Estimation of the clinically diagnosed proportion of sleep apnea syndrome in middle-aged men and women. Sleep (1997) 20(9):705–6. 9406321 10.1093/sleep/20.9.705

[10] Wolfe RM Pomerantz J Miller DE Weiss-Coleman R Solomonides T. Obstructive sleep apnea: preoperative screening and postoperative care. J Am Board Fam Med (2016) 29(2):263–75. 10.3122/jabfm.2016.02.150085 26957384

[11] Abrishami A Khajehdehi A Chung F. A systematic review of screening questionnaires for obstructive sleep apnea. Can J Anaesth (2010) 57(5):423–38. 10.1007/s12630-010-9280-x 20143278

[12] Gali B Whalen FX Schroeder DR Gay PC Plevak DJ. Identification of patients at risk for postoperative respiratory complications using a preoperative obstructive sleep apnea screening tool and postanesthesia care assessment. Anesthesiology (2009) 110(4):869–77. 10.1097/ALN.0b013e31819b5d70 19293694

[13] Xara D Mendonca J Pereira H Santos A Abelha FJ [Adverse respiratory events after general anesthesia in patients at high risk of obstructive sleep apnea syndrome]. Rev Bras Anestesiol (2015) 65(5):359–66. 10.1016/j.bjan.2014.02.008 26363693

[14] Toh ST Han HJ Tay HN Kiong KL. Transoral robotic surgery for obstructive sleep apnea in Asian patients: a Singapore sleep centre experience. JAMA Otolaryngol Head Neck Surg (2014) 140(7):624–9. 10.1001/jamaoto.2014.926 24921220

[15] Montravers P Dureuil B Desmonts JM. Effects of i.v. midazolam on upper airway resistance. Br J Anaesth (1992) 68(1):27–31. 10.1093/bja/68.1.27 1739562

[16] Gupta RM Parvizi J Hanssen AD Gay PC. Postoperative complications in patients with obstructive sleep apnea syndrome undergoing hip or knee replacement: a case-control study. Mayo Clin Proc (2001) 76(9):897–905. 10.4065/76.9.897 11560300

[17] Singh M Liao P Kobah S Wijeysundera DN Shapiro C Chung F Proportion of surgical patients with undiagnosed obstructive sleep apnoea. Br J Anaesth (2013) 110(4):629–36. 10.1093/bja/aes465 23257990

[18] Hai F Porhomayon J Vermont L Frydrych L Jaoude P El-Solh AA. Postoperative complications in patients with obstructive sleep apnea: a meta-analysis. J Clin Anesth (2014) 26(8):591–600. 10.1016/j.jclinane.2014.05.010 25439403

[19] Lee W Nagubadi S Kryger MH Mokhlesi B. Epidemiology of obstructive sleep apnea: a population-based perspective. Expert Rev Respir Med (2008) 2(3):349–64. 10.1586/17476348.2.3.349 19690624 PMC2727690

[20] Lavie L Oxidative stress in obstructive sleep apnea and intermittent hypoxia – revisited – the bad ugly and good: implications to the heart and brain. Sleep Med Rev (2015) 20:27–45. 10.1016/j.smrv.2014.07.003 25155182

[21] Cadby G McArdle N Briffa T Hillman DR Simpson L Knuiman M Severity of OSA is an independent predictor of incident atrial fibrillation hospitalization in a large sleep-clinic cohort. Chest (2015) 148(4):945–52. 10.1378/chest.15-0229 25927872

[22] Somers VK White DP Amin R Abraham WT Costa F Culebras A Sleep apnea and cardiovascular disease: an American Heart Association/American College of Cardiology Foundation Scientific Statement from the American Heart Association Council for High Blood Pressure Research Professional Education Committee, Council on Clinical Cardiology, Stroke Council, and Council on Cardiovascular Nursing. In Collaboration with the National Heart, Lung, and Blood Institute National Center on Sleep Disorders Research (National Institutes of Health). Circulation (2008) 118(10):1080–111. 10.1161/CIRCULATIONAHA.107.189375 18725495

[23] Lockhart EM Willingham MD Abdallah AB Helsten DL Bedair BA Thomas J Obstructive sleep apnea screening and postoperative mortality in a large surgical cohort. Sleep Med (2013) 14(5):407–15. 10.1016/j.sleep.2012.10.018 23499198 PMC4575681

[24] Peker Y Hedner J Norum J Kraiczi H Carlson J. Increased incidence of cardiovascular disease in middle-aged men with obstructive sleep apnea: a 7-year follow-up. Am J Respir Crit Care Med (2002) 166(2):159–65. 10.1164/rccm.2105124 12119227

[25] Xie J Sert Kuniyoshi FH Covassin N Singh P Gami AS Wang S Nocturnal hypoxemia due to obstructive sleep apnea is an independent predictor of poor prognosis after myocardial infarction. J Am Heart Assoc (2016) 5(8). 10.1161/JAHA.115.003162 PMC5015271 27464791

[26] Chung F Liao P Yang Y Andrawes M Kang W Mokhlesi B Postoperative sleep-disordered breathing in patients without preoperative sleep apnea. Anesth Analg (2015) 120(6):1214–24. 10.1213/ANE.0000000000000774 25988633

[27] Dette F Cassel W Urban F Zoremba M Koehler U Wulf H Occurrence of rapid eye movement sleep deprivation after surgery under regional anesthesia. Anesth Analg (2013) 116(4):939–43. 10.1213/ANE.0b013e3182860e58 23460574

[28] Mulier JP. Perioperative opioids aggravate obstructive breathing in sleep apnea syndrome: mechanisms and alternative anesthesia strategies. Curr Opin Anaesthesiol (2016) 29(1):129–33. 10.1097/ACO.0000000000000281 26595546

[29] Kaw R Perioperative complications in patients with obstructive sleep apnea. Curr Anesthesiol Rep (2013) 4(1):37–41. 10.1007/s40140-013-0044-3

[30] Gross JB Bachenberg KL Benumof JL Caplan RA Connis RT Cote CJ Practice guidelines for the perioperative management of patients with obstructive sleep apnea: a report by the American Society of Anesthesiologists Task Force on Perioperative Management of patients with obstructive sleep apnea. Anesthesiology (2006) 104(5):1081–93; quiz 117–8. 10.1097/00000542-200605000-00026 16645462

[31] Pope J McBride J Consultation with the specialist: respiratory failure in children. Pediatr Rev (2004) 25(5):160–7. 10.1542/pir.25-5-160 15121907

[32] Mendes Tde A Andreoli PB Cavalheiro LV Talerman C Laselva C. Adjustment of oxygen use by means of pulse oximetry: an important tool for patient safety. Einstein (Sao Paulo) (2010) 8(4):449–55. 10.1590/S1679-45082010AO1377 26760328

[33] Pedersen T Nicholson A Hovhannisyan K Moller AM Smith AF Lewis SR Pulse oximetry for perioperative monitoring. Cochrane Database Syst Rev (2014) 3:CD002013 10.1002/14651858.CD002013.pub3 PMC6464860 24638894

[34] Ayas N Bergstrom LR Schwab TR Narr BJ. Unrecognized severe postoperative hypercapnia: a case of apneic oxygenation. Mayo Clin Proc (1998) 73(1):51–4. 10.1016/S0025-6196(11)63619-7 9443679

[35] Levy P Pepin JL Deschaux-Blanc C Paramelle B Brambilla C. Accuracy of oximetry for detection of respiratory disturbances in sleep apnea syndrome. Chest (1996) 109(2):395–9. 10.1378/chest.109.2.395 8620711

[36] Chung F Liao P Elsaid H Islam S Shapiro CM Sun Y. Oxygen desaturation index from nocturnal oximetry: a sensitive and specific tool to detect sleep-disordered breathing in surgical patients. Anesth Analg (2012) 114(5):993–1000. 10.1213/ANE.0b013e318248f4f5 22366847

[37] Qadeer MA Vargo JJ Dumot JA Lopez R Trolli PA Stevens T Capnographic monitoring of respiratory activity improves safety of sedation for endoscopic cholangiopancreatography and ultrasonography. Gastroenterology (2009) 136(5):1568–76; quiz 819–20. 10.1053/j.gastro.2009.02.004 19422079

[38] Kjorven M Dunton D Milo R Gerein L Bedside capnography: better management of surgical patients with obstructive sleep apnea. Can Nurse (2011) 107(9):24–6. 22132609

[39] Hillman DR Platt PR Eastwood PR. The upper airway during anaesthesia. Br J Anaesth (2003) 91(1):31–9. 10.1093/bja/aeg126 12821563

[40] Hillman DR Loadsman JA Platt PR Eastwood PR. Obstructive sleep apnoea and anaesthesia. Sleep Med Rev (2004) 8(6):459–71. 10.1016/j.smrv.2004.07.002 15556378

[41] Kasuya Y Akca O Sessler DI Ozaki M Komatsu R. Accuracy of postoperative end-tidal Pco2 measurements with mainstream and sidestream capnography in non-obese patients and in obese patients with and without obstructive sleep apnea. Anesthesiology (2009) 111(3):609–15. 10.1097/ALN.0b013e3181b060b6 19672177

[42] Gaucher A Frasca D Mimoz O Debaene B. Accuracy of respiratory rate monitoring by capnometry using the Capnomask(R) in extubated patients receiving supplemental oxygen after surgery. Br J Anaesth (2012) 108(2):316–20. 10.1093/bja/aer383 22157953

[43] Leonard PA Douglas JG Grubb NR Clifton D Addison PS Watson JN. A fully automated algorithm for the determination of respiratory rate from the photoplethysmogram. J Clin Monit Comput (2006) 20(1):33–6. 10.1007/s10877-005-9007-7 16532280

[44] Addison PS Watson JN Mestek ML Ochs JP Uribe AA Bergese SD. Pulse oximetry-derived respiratory rate in general care floor patients. J Clin Monit Comput (2015) 29(1):113–20. 10.1007/s10877-014-9575-5 24796734 PMC4309914

[45] Knorr-Chung BR McGrath SP Blike GT. Identifying airway obstructions using photoplethysmography (PPG). J Clin Monit Comput (2008) 22(2):95–101. 10.1007/s10877-008-9110-7 18219579

[46] Romem A Romem A Koldobskiy D Scharf SM. Diagnosis of obstructive sleep apnea using pulse oximeter derived photoplethysmographic signals. J Clin Sleep Med (2014) 10(3):285–90. 10.5664/jcsm.3530 24634626 PMC3927434

[47] Nilsson LM. Respiration signals from photoplethysmography. Anesth Analg (2013) 117(4):859–65. 10.1213/ANE.0b013e31828098b2 23449854

[48] Karmakar C Khandoker A Penzel T Schobel C Palaniswami M. Detection of respiratory arousals using photoplethysmography (PPG) signal in sleep apnea patients. IEEE J Biomed Health Inform (2014) 18(3):1065–73. 10.1109/JBHI.2013.2282338 24108482

[49] Penzel T Blau A Garcia C Schobel C Sebert M Baumann G [Diagnosis of sleep disordered breathing using portable methods]. Pneumologie (2013) 67(2):112–7. 10.1055/s-0032-1325943 23247596

[50] Meredith DJ Clifton D Charlton P Brooks J Pugh CW Tarassenko L. Photoplethysmographic derivation of respiratory rate: a review of relevant physiology. J Med Eng Technol (2012) 36(1):1–7. 10.3109/03091902.2011.638965 22185462

[51] Knorr BR McGrath SP Blike GT. Using a generalized neural network to identify airway obstructions in anesthetized patients postoperatively based on photoplethysmography. Conf Proc IEEE Eng Med Biol Soc (2006) Suppl:6765–8. 10.1109/IEMBS.2006.260942 17959507

[52] Voscopoulos CJ MacNabb CM Freeman J Galvagno SM Jr Ladd D George E. Continuous noninvasive respiratory volume monitoring for the identification of patients at risk for opioid-induced respiratory depression and obstructive breathing patterns. J Trauma Acute Care Surg (2014) 77(3 Suppl 2):S208–15. 10.1097/TA.0000000000000400 25159358

[53] Ebert TJ Middleton AH Makhija N Ventilation monitoring during moderate sedation in GI patients. J Clin Monit Comput (2017) 31(1):53–7. 10.1007/s10877-015-9809-1 26628270

[54] Williams GW II George CA Harvey BC Freeman JE A comparison of measurements of change in respiratory status in spontaneously breathing volunteers by the exspiron noninvasive respiratory volume monitor versus the capnostream capnometer. Anesth Analg (2017) 124(1):120–6. 10.1213/ANE.0000000000001395 27384980

[55] Voscopoulos C Theos K Tillmann Hein HA George E. A risk stratification algorithm using non-invasive respiratory volume monitoring to improve safety when using post-operative opioids in the PACU. J Clin Monit Comput (2016). 10.1007/s10877-016-9841-9 26894592

[56] Voscopoulos CJ MacNabb CM Brayanov J Qin L Freeman J Mullen GJ The evaluation of a non-invasive respiratory volume monitor in surgical patients undergoing elective surgery with general anesthesia. J Clin Monit Comput (2015) 29(2):223–30. 10.1007/s10877-014-9596-0 25037938

[57] Voscopoulos C Brayanov J Ladd D Lalli M Panasyuk A Freeman J. Special article: evaluation of a novel noninvasive respiration monitor providing continuous measurement of minute ventilation in ambulatory subjects in a variety of clinical scenarios. Anesth Analg (2013) 117(1):91–100. 10.1213/ANE.0b013e3182918098 23733842

[58] Voscopoulos C Ladd D Campana L George E. Non-invasive respiratory volume monitoring to detect apnea in post-operative patients: case series. J Clin Med Res (2014) 6(3):209–14. 10.14740/jocmr1718w 24734148 PMC3985564

[59] Murphy GS Szokol JW Marymont JH Greenberg SB Avram MJ Vender JS. Residual neuromuscular blockade and critical respiratory events in the postanesthesia care unit. Anesth Analg (2008) 107(1):130–7. 10.1213/ane.0b013e31816d1268 18635478

[60] Goucham AB Coblijn UK Hart-Sweet HB de Vries N Lagarde SM van Wagensveld BA. Routine postoperative monitoring after bariatric surgery in morbidly obese patients with severe obstructive sleep apnea: ICU admission is not necessary. Obes Surg (2016) 26(4):737–42. 10.1007/s11695-015-1807-3 26210193

